# Exploring Application of Smart Companion Robots to Facilitate Faith-Based Ministries for Older Homebound Adults

**DOI:** 10.1093/geroni/igaa057.1009

**Published:** 2020-12-16

**Authors:** Christianah Adebayo, Alex Bishop

**Affiliations:** Oklahoma State University, Stillwater, Oklahoma, United States

## Abstract

This study examined perceptions regarding application of smart robot companions to facilitate homebound religious ministry programming to older adults. A total of N= 7 participants consisting of ministers, pastoral volunteers, and older adult members from Methodist, Presbyterian, and Roman Catholic traditions engaged in a 60-minute focus group. One key goal was to address the question: If you could design a robot for ministry to homebound older adults what would you want it to do? All responses were recorded, transcribed and coded for thematic content. Three core themes emerged relative to spiritual privacy, prospective intervention, and inclusive monitoring. Relative to spiritual privacy, participants expressed concern regarding robotic ability to maintain the spiritual privacy of the older homebound adult, especially when engaged in faith-based behaviors such as private prayer, confession, and pastoral counseling. Second, participants suggested that a robot companion serve as a memory aide for older homebound members. In particular, participants acknowledged a need for robot intervention relative to sending alerts concerning prayer requests, reminders of upcoming church-related events, and notices concerning future homebound ministry visitations. Finally, participants indicated an interest for the robot to actively monitor community inclusion, noting that many older homebound adults are left out of weekly church-related services or events and experience seclusion, yet most “retain a desire be included and remain an integral part of their faith community.” Results will be used to present a conceptual model for a smart robotic faith assistant designed for delivery of homebound ministries to older adults.

